# Molecular epidemiology and genetic analysis of HCV infection in Cyprus within an intravenous drug user study cohort

**DOI:** 10.1186/1742-4690-9-S1-P51

**Published:** 2012-05-25

**Authors:** Demetris C Iacovides, Johana Hezka, Natasa Savvopoulou, Athos Chrysanthou, Leontios G Kostrikis

**Affiliations:** 1 University, Nicosia, Cyprus

## Introduction

The HCV genome is highly heterogeneous, owing to the high mutation rate of RNA viruses. There are seven HCV genotypes, with numerous subtypes and viral quasispecies. The prevalence and distribution of HCV genotypes varies globally, and strains show highly variable sensitivity to available therapeutics. Thus, genetic and epidemiological studies of hepatitis C infection are significant, especially within high-risk cohorts where viral evolution can be rapid and unpredictable.

## Materials and methods

We collected blood samples from intravenous drug users (IVDU) and performed ELISAs to determine HCV positivity. RNA extraction, reverse transcription PCR and DNA sequencing were performed in the Core-E1 and NS5B regions. Strain subtyping was performed using the Oxford HCV subtyping tool v2.0. Phylogenetic analysis was done by aligning and comparing the sequences of both regions to reference strains from the Los Alamos database, using the neighbor-joining method and the Kimura two-parameter distance estimation approach in MEGA v4. The reliability of the phylogenetic clustering was evaluated using bootstrap analysis with 1,000 replicates, and bootstrap values above 70 were considered sufficient for subtype assignment.

## Results

21 out of 64 research subjects (32.8%) were positive for HCV, a percentage much lower than the global average (60%) amongst IVDUs. All individuals were infected with genotypes 3a (62%), or 1b (32%), and no unique recombinants were identified. Interestingly, none of the subjects were infected with the 1a strain, a genotype that is highly associated with intravenous drug use, and is also prevalent in the general Cypriot population. In contrast, 38% of the subjects were infected with strain 1b, which is fairly uncommon amongst IVDUs. Finally, we observed three small clusters within the IVDU group, suggesting possible sharing of injecting equipment.

## Conclusions

HCV infection within IVDUs in Cyprus is polyphyletic, with high genetic heterogeneity, as seen by the limited clustering within this group. Absence of large clusters also suggests that sharing of injecting equipment is uncommon. HCV prevalence amongst IVDUs is significantly lower than the global average, and only circulation of subtypes 1b and 3a is observed, in contrast to the general population where all genotypes are present.

